# Antimicrobial stewardship challenges during the COVID-19 pandemic: implications of antibiotic overuse and antimicrobial resistance

**DOI:** 10.2478/aiht-2026-77-4130

**Published:** 2026-09-25

**Authors:** Marija Špelić, Vladimir Krajinović

**Affiliations:** Health Institution Pharmacies Župančić, Popovača, Croatia; Dr Fran Mihaljević Hospital for Infectious Diseases, Zagreb, Croatia

**Keywords:** antimicrobial resistance, antimicrobial stewardship, bacterial co-infection, One Health, antimikrobna rezistencija, bakterijska koinfekcija, *Jedno zdravlje*, upravljanje upotrebom antibiotika

## Abstract

The coronavirus disease 2019 (COVID-19) pandemic significantly influenced antimicrobial prescribing practices worldwide, raising concerns about antimicrobial resistance (AMR). This narrative review assesses the relationship between COVID-19 and antibiotic use, focusing on bacterial co-infections, prescribing patterns, long-term implications for AMR, and the role of multidisciplinary antimicrobial and diagnostic stewardship, preventive strategies, and the One Health approach in reducing disproportionate antibiotic exposure. To this end we ran a subject search across PubMed/MEDLINE, Scopus, Web of Science, the Cochrane Database of Systematic Reviews, Ovid, and Google Scholar for studies selected according to predefined inclusion and exclusion criteria. Despite relatively low rates of confirmed bacterial co-infections, antibiotic use remained disproportionately high, particularly during the early stages of the pandemic, highlighting challenges in clinical decision-making under diagnostic uncertainty. Excessive and inappropriate antibiotic prescribing may have contributed to the global AMR burden. Strengthening antimicrobial stewardship programmes, improving diagnostic accuracy and promoting evidence-based prescribing are therefore essential. Multidisciplinary antimicrobial stewardship interventions, rapid molecular diagnostics and vaccination-associated reductions in antibiotic prescribing emerge as complementary strategies for limiting unnecessary antimicrobial exposure, while the environmental consequences of antibiotic use further emphasise the relevance of the One Health approach. Continued surveillance and research are needed to better understand the long-term impact of the pandemic on AMR.

## Abstract (hr)

Pandemija koronavirusne bolesti (COVID-19) značajno je utjecala na praksu propisivanja antimikrobnih lijekova širom svijeta, izazivajući zabrinutost zbog antimikrobne rezistencije (AMR). Cilj je ovoga preglednog rada ispitati odnos između bolesti COVID-19 i uporabe antibiotika, s naglaskom na učestalost bakterijskih koinfekcija, obrasce propisivanja i na moguće dugoročne posljedice za AMR. Istaknuta je uloga multidisciplinarnog upravljanja antimikrobnom terapijom, racionalnog upravljanja dijagnostičkim postupcima, preventivnih strategija i pristupa *Jednog zdravlja*. Pregled literature proveden je pretraživanjem baza PubMed/MEDLINE, Scopus, Web of Science, Cochrane Database of Systematic Reviews, Ovid i Google Scholar, uz unaprijed određene kriterije uključivanja i isključivanja. Unatoč relativno niskim stopama potvrđenih bakterijskih koinfekcija, uporaba antibiotika bila je neproporcionalno visoka, osobito tijekom ranih faza pandemije, što je naglasilo izazove kliničkog odlučivanja u uvjetima dijagnostičke nesigurnosti. Jačanje programa upravljanja antimikrobnom terapijom, poboljšanje dijagnostičke točnosti i propisivanje temeljeno na dokazima ključni su za smanjenje nepotrebne izloženosti antibioticima. Multidisciplinarne AMS intervencije, brza molekularna dijagnostika i smanjenje propisivanja antibiotika povezano s cijepljenjem pokazali su se kao komplementarne strategije, dok su okolišne posljedice primjene antibiotika dodatno istaknule važnost pristupa *Jednog zdravlja*. Potrebni su kontinuirano praćenje i daljnja istraživanja radi boljeg razumijevanja dugoročnog utjecaja pandemije na AMR.

The COVID-19 pandemic, caused by SARS-CoV-2, has resulted in more than 770 million confirmed cases and over seven million deaths worldwide (1). Beyond the direct burden of acute viral infection, the pandemic has revealed a growing and often under-recognised threat of antimicrobial resistance (AMR). The World Health Organization identified AMR as a global health priority, with the Global Action Plan adopted in 2015 and AMR explicitly recognised as a priority for 2020, warning that disproportionate or unnecessary antimicrobial use threatens the foundation of modern medicine (2, 3).

The early phase of the pandemic was characterised by profound clinical uncertainty, concern regarding rapid disease progression, and the absence of effective antiviral therapy. In this context, antibiotics were often prescribed empirically, despite COVID-19 being a viral disease. This practice was partly driven by assumptions regarding frequent bacterial co-infections, extrapolated from influenza and previous coronavirus outbreaks such as severe acute respiratory syndrome (SARS) and Middle East respiratory syndrome (MERS) (3).

Subsequent systematic reviews demonstrated that bacterial co-infection at hospital admission occurs in only 3–8 % of patients (4,5,6,7,8). Yet, antibiotic prescribing increased substantially, particularly of broad-spectrum agents and macrolides (9). This discrepancy between clinical need and prescribing practice may have contributed to antimicrobial selection pressure and the emergence and spread of AMR. In addition, the pandemic disrupted infection prevention and control practices, strained healthcare systems, and limited access to microbiological diagnostics. Conversely, it also stimulated innovation in antimicrobial stewardship (AMS), including improved diagnostic algorithms and optimisation of antimicrobial prescribing. This narrative review aims to synthesise current evidence on antibiotic use during the COVID-19 pandemic, evaluate its impact on AMR, and assess the role of AMS programmes in mitigating long-term public health consequences.

## METHODS

To this end we ran a structured literature search in accordance with the established principles for biomedical reviews. Scientific databases including PubMed/MEDLINE, Scopus, Web of Science, Cochrane Database of Systematic Reviews, Ovid, and Google Scholar were searched for studies published between January 2020 and June 2026.

Search terms included combinations of “COVID-19”, “SARS-CoV-2”, “bacterial co-infection”, “secondary infection”, “antibiotic use”, “antimicrobial resistance”, “antimicrobial stewardship”, and „One Health“. Eligible studies included randomised controlled trials, cohort studies, systematic reviews, meta-analyses, and relevant clinical guidelines addressing antibiotic use in COVID-19, prevalence of bacterial co-infections, AMR trends, and AMS interventions.

The literature search identified 344 records. After removal of duplicates, 322 records were screened by title and abstract, and 232 excluded because they did not address antibiotic use, antimicrobial stewardship, antimicrobial resistance, or bacterial co-infections in the context of COVID-19. Full texts of the remaining 90 articles were assessed for eligibility, of which 53 were excluded over insufficient data on antibiotic prescribing, lack of AMS or AMR outcomes, or limited relevance to the One Health and vaccination-related objectives.

**Figure 1:**
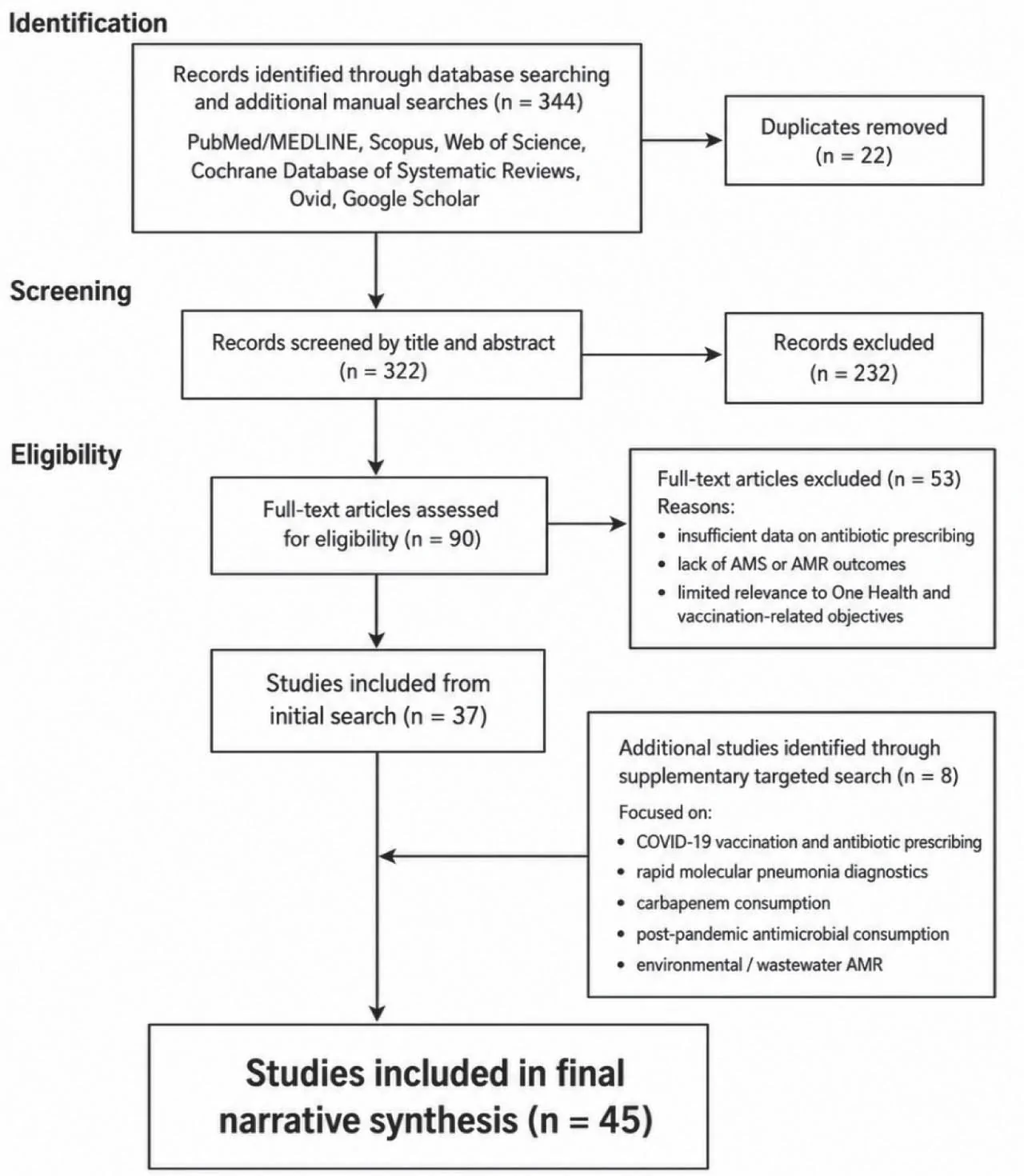
The PRISMA flow diagram of study selection

Following the initial screening and selection, we ran a supplementary targeted search focused on COVID-19 vaccination and antibiotic prescribing, rapid molecular pneumonia diagnostics, carbapenem consumption, post-pandemic antimicrobial consumption, and environmental/wastewater aspects of antimicrobial resistance to identify additional studies relevant to specific issues raised during the review process, resulting in the inclusion of eight additional studies in the final synthesis, totalling 45 studies. Although this study was designed as a narrative review, the literature search and study selection process were conducted and reported using relevant elements of the PRISMA 2020 framework to enhance transparency of literature identification and selection and reproducibility (10) (Figure 1).

## RESULTS AND DISCUSSION

### Patterns of antibiotic prescribing during COVID-19

Since its emergence in Wuhan in late 2019, SARS-CoV-2 rapidly spread worldwide and resulted in substantial global morbidity and mortality. Global excess mortality associated with the COVID-19 pandemic was estimated to about 18.2 million deaths between January 2020 and December 2021, reflecting both direct and indirect effects of the pandemic (11). Despite the viral aetiology of COVID-19, antibiotics were prescribed widely throughout the pandemic. Early COVID-19 recommendations promoted empirical antibiotic therapy because of concerns regarding bacterial co-infection and superinfection (12). Diagnostic uncertainty, overlapping clinical manifestations between viral and bacterial pneumonia, and early hypotheses regarding potential antiviral effects of macrolides further contributed to extensive empirical antibiotic prescribing in both hospital and community settings (13, 14). However, subsequent large, randomised trials demonstrated no clinical benefit of azithromycin-based regimens in COVID-19 patients (15).

Most empirical antibiotic regimens targeted community-acquired pneumonia and primarily consisted of ceftriaxone combined with azithromycin (16). Frequently prescribed antibiotics also included amoxicillin-clavulanate, piperacillin-tazobactam, macrolides, carbapenems, cephalosporins and fluoroquinolones (17). Carbapenem consumption increased markedly in some hospital settings. In one centre, it increased from a baseline of 67.1 to 142.9 defined daily doses (DDD) per 1,000 patient-days during the COVID-19 pre-intervention period, while intensive-care unit (ICU) consumption increased from 125.7 to 240.8 DDD per 1,000 patient-days. Following implementation of targeted stewardship measures, carbapenem consumption decreased by 49.5 % hospital-wide and by 36 % in ICUs (18). High-risk antibiotics associated with *Clostridioides difficile* infection (CDI), including clindamycin, fluoroquinolones, and third-generation cephalosporins, were increasingly prescribed during the pandemic (12). Broad-spectrum antibiotic prescribing became common despite the viral nature of the COVID-19 (19). Importantly, early antibiotic administration did not slow down disease progression, shorten the length of hospital stays, or lower mortality in most hospitalised COVID-19 patients without confirmed bacterial infection (17).

The substantial increase in antibiotic prescribing during the pandemic challenged antimicrobial stewardship programmes and raised concerns regarding the emergence and spread of multidrug-resistant organisms. Regional differences in prescribing practices were also observed. North America reported the highest prevalence of antibiotic use among COVID-19 patients (68.84 %, 95 % CI 62.27–75.05), followed by Europe (60.01 %, 95 % CI 25.50–89.67), whereas Asia reported the lowest prevalence (40.81 %, 95 % CI 7.75–79.65) (14). Although Europe experienced lower overall antibiotic consumption in 2020, likely due to reduced medical treatment activity and social distancing, antibiotic prescribing among hospitalised COVID-19 patients remained high (14). In the United States, outpatient antibiotic prescription also declined substantially in 2020, particularly for commonly used antibiotics such as amoxicillin, amoxicillin-clavulanate, doxycycline, and azithromycin (7). Antibiotic consumption patterns also changed following the acute pandemic period. After the marked reductions observed in 2020 and 2021, community antibiotic consumption in the EU/EEA rebounded in 2022. Total EU antibacterial consumption reached 19.4 DDD per 1,000 inhabitants per day, representing a significant increase compared to 2020–2021, although remaining approximately 2.5 % below the 2019 baseline (20).

The pandemic also altered broader antibiotic utilisation and resistance patterns. Longer hospital stays were observed during the pandemic (3.86 days in 2019 v 4.29 days in 2020), accompanied by greater use of duplicate anaerobic therapy (4.58 % in 2019 v 5.71 % in 2020) and greater prescribing of antibiotics for viral and fungal infections (17.08 % in 2019 v 22.38 % in 2020) (21). In addition, erroneous empirical antibiotic prescribing increased during the pandemic, while antimicrobial resistance (AMR) challenges persisted despite modest improvements in stewardship practices (21). These findings emphasise the need for strengthened antimicrobial stewardship programmes and continued surveillance to mitigate the long-term consequences of inappropriate antibiotic prescribing and AMR development.

Frequent empirical prescribing of antibiotics stood out against the relatively low prevalence of documented bacterial infections. In a comparative cohort study (22), bacterial infections were identified in only 8 % of hospitalised patients with COVID-19, compared to 13 % with non-COVID-19 pneumonia, and most bacterial infections among COVID-19 patients were nosocomial rather than community-acquired. The overwhelming number of hospitalised COVID-19 patients further amplified antibiotic consumption. One study (23) demonstrated a quadrupling of ceftriaxone use and a doubling of azithromycin use during the initial stages of the pandemic. One systematic review and meta-analysis (24) reported a pooled prevalence of bacterial co-infection of 8 % and bacterial superinfection of 20 %, while antibiotics were administered in 98 % of studies reporting antibiotic use. In a stewardship-focused intervention study including 833 hospitalised patients (25), antibiotics were prescribed to 438 (53 %) patients at enrolment. Among 429 patients allocated to the intervention group, 203 (47 %) received at least one antibiotic at enrolment, while 32 (7 %) more were prescribed antibiotics later during hospitalisation. A total of 301 prospective audit and feedback events were conducted, including 235 initial audits and 66 follow-up audits. Ceftriaxone (216 prescriptions) and azithromycin (167 prescriptions) were the two most audited antibiotics, in line with local recommendations for the management of hospitalised patients with community-acquired pneumonia. These findings are consistent with additional cohort and meta-analytic evidence demonstrating low rates of bacterial co-infection alongside substantial antibiotic over-prescription among hospitalised COVID-19 patients (26, 27). The main patterns of inappropriate antibiotic use, including route of administration, duration-related problems, and key stewardship concerns, are summarised in Table 1.

**Table 1:** Patterns of disproportionate antibiotic use during the COVID-19 pandemic

**Antibiotic class**	**Common agents reported**	**Route**	**Duration-related problem**	**Type of misuse/AMS concern**	**Ref.**
Cephalosporins	Ceftriaxone and third-generation cephalosporins	IV	Treatment >7 days despite negative cultures, IV therapy >48 h without microbiological confirmation	Empirical treatment despite low bacterial co-infection rates, increased CDI risk, frequent use in ceftriaxone-azithromycin regimens	12, 25, 28
Macrolides	Azithromycin	PO/IV	Empirical use often continued despite lack of proven benefit	Presumed antiviral effect, unnecessary use and AMR selection pressure	23, 25
Carbapenems	Meropenem	IV	Broad-spectrum escalation during hospitalisation	Selection of MDR Gram-negative organisms, need for AMS restriction policies	17
Fluoroquinolones	Levofloxacin; moxifloxacin	PO/IV	Prolonged use despite negative microbiological findings	CDI risk, AMR selection pressure and unnecessary broad-spectrum coverage	12, 17, 28
Penicillins	Amoxicillin; ampicillin	PO/IV	Empirical prescribing without microbiological confirmation	Use for viral respiratory illness and low-probability bacterial infection	17, 19
β-lactam/β-lactamase inhibitor combinations	Piperacillin/tazobactam	IV	Increased use and escalation to broad-spectrum therapy	Selection pressure for MDR Gram-negative bacteria, need for reassessment and de-escalation	17, 19, 21
Aminopenicillin/β-lactamase inhibitor combinations	Amoxicillin/clavulanate	PO/IV	Continued use despite low likelihood of bacterial co-infection	Empirical respiratory infection treatment without confirmed bacterial infection	17

AMR – antimicrobial resistance; AMS – antimicrobial stewardship; CDI – *Clostridioides difficile* infection; IV – intravenous; MDR – multidrug-resistant; PO – peroral

### Bacterial co-infections and mismatch with antibiotic use

Current evidence suggests that bacterial co-infection rates in patients with COVID-19 were substantially lower than those observed during previous viral pandemics. Direct pre-pandemic comparisons support this observation. In a retrospective cohort study (22) comparing patients hospitalised with non-COVID-19 pneumonia in 2019 with patients admitted with COVID-19 and pulmonary infiltrates in 2020, microbiologically confirmed bacterial infections were identified in 13 % and 8 %, respectively (P<0.001). During the 2009 influenza A (H1N1) pandemic, bacterial pneumonia contributed to up to 55 % of fatalities (14). In contrast, one meta-analysis (26) conducted during the COVID-19 pandemic demonstrated that bacterial co-infections at hospital admission were relatively uncommon, with reported prevalence rates ranging between 3 % and 5 %, whereas secondary bacterial infections occurred more frequently among critically ill and mechanically ventilated patients. Despite these low rates of confirmed bacterial co-infection, antibiotics were prescribed to 60–75 % of hospitalised COVID-19 patients, constituting a substantial mismatch between microbiological evidence and prescribing practices. Although recognition gradually emerged that bacterial co-infections were uncommon in COVID-19, antibiotics continued to be prescribed at disproportionately high rates (25). Bacterial co-infection and secondary infection were also associated with adverse clinical outcomes. One systematic review and meta-analysis (24) found that COVID-19 patients with co-infection or superinfection had more than three times higher odds of death than patients with SARS-CoV-2 infection alone (OR 3.31; 95 % CI 1.82–5.99).

Several factors contributed to this discrepancy between confirmed bacterial infection and empirical antibiotic use such as pandemic-related healthcare burden, diagnostic uncertainty, concerns about bacterial superinfection, and disruption of established AMS activities (27). The central stewardship challenge was not the complete absence of bacterial infections, but rather the difficulty in distinguishing early viral pneumonia from true bacterial co-infection, particularly in severely ill patients, leading to extensive empirical antibiotic prescribing without microbiological confirmation (17).

In addition, one systematic review (28) demonstrated inadequate reassessment of empirical antibiotic therapy during hospitalisation and identified opportunities for improvement to address issues such as prolonged antibiotic treatment exceeding seven days in 37 % of patients receiving empirical antibiotics despite negative cultures, intravenous therapy exceeding 48 h, and reluctance to switch to oral therapy, ranging from only 9.9 % to 40.5 %, depending on reports covered by this review. It also revealed that antibiotic prescribing increased from 60.1 % at admission to 72.3 % after seven days of hospitalisation, even though bacterial co-infection was confirmed in only 1.2 % of patients.

### Impact on antimicrobial resistance dynamics

The COVID-19 pandemic created a unique and highly complex epidemiological and clinical environment involving extraordinary pressure on healthcare systems, altered population behaviour, and substantial deviations in antibiotic prescribing practices. Under such circumstances, certain aspects of the pandemic response may have accelerated AMR development, while others temporarily exerted protective effects (29). The widespread empirical antibiotic prescribing in the early stages of the pandemic generated considerable antimicrobial selection pressure and raised concerns regarding long-term AMR consequences in hospital settings. In fact, the emerging evidence indicates increasing resistance to critically important antibiotics, particularly carbapenems and colistin (6, 29). Secondary bacterial infections were observed predominantly among critically ill and mechanically ventilated patients and were frequently caused by nosocomial multidrug-resistant pathogens, including *Acinetobacter baumannii*, *Klebsiella pneumoniae*, and *Pseudomonas aeruginosa* (26, 30). In some healthcare settings, increased incidence of carbapenem-resistant *Enterobacterales* was also reported (31). Collectively, these findings suggest that pandemic-related prescribing practices, prolonged hospitalisation, and increased ICU exposure may have accelerated the emergence and spread of multidrug-resistant organisms.

Besides, the pandemic disrupted infection prevention and control (IPC) activities and AMS programmes worldwide. Healthcare systems faced severe staff shortages, overwhelming workloads, and limited diagnostic capacity, all of which contributed to delayed microbiological confirmation, excessive empirical antibiotic use, and transmission of multidrug-resistant organisms within healthcare facilities (27, 32). In a cohort of hospitalised COVID-19 patients with documented bacterial or fungal co-infection mortality reached 57 %, highlighting the poor prognosis associated with clinically significant secondary infections (33). The pandemic therefore highlighted the critical importance of maintaining diagnostic capacity, microbiological surveillance, and rational antibiotic prescribing, reserved for confirmed or strongly suspected bacterial infections, even during public health emergencies (23, 32).

### Structure and role of AMS teams during the pandemic

For multiple reasons, AMS became “a victim” of COVID-19, as major disruptions in clinical service delivery, global antibiotic supply chains, and the tendency toward overtreatment with antibiotics weakened established stewardship practices (19), particularly in regions where stewardship programmes were still developing and resource limitations already existed (14). The pandemic also disrupted ongoing antimicrobial stewardship programmes in high-income healthcare systems, highlighting the vulnerability of stewardship infrastructure (27).

Despite these challenges, AMS programmes played a critical role in optimising antibiotic use during the COVID-19 pandemic. Effective stewardship strategies included infectious disease specialist participation in clinical rounds, biweekly review of ongoing antibiotic therapy, dissemination of COVID-19 treatment guidelines through mobile applications, and prospective weekly audits (17). One carbapenem-focused AMS intervention utilised pharmacy alerts to infectious disease specialists following carbapenem prescription, with specialist consultation occurring within 72 h. This intervention reduced carbapenem use from 4.1 % to 2.3 % and was associated with lower in-hospital mortality and improved treatment success rates (17). Predictive models combining white blood cell count, procalcitonin levels, and comorbidity burden were also proposed as useful tools for identifying patients at low risk of bacterial co-infection and supporting rational antibiotic prescribing (17).

Prospective audit and feedback emerged as one of the most effective stewardship interventions during the pandemic and was shown to be safe and effective in optimising and reducing antibiotic use among adults hospitalised with COVID-19 (25). Chen et al. (25) reported high physician acceptance rate of stewardship recommendations (84 %) including discontinuation of antibiotic therapy when bacterial infection was not suspected or confirmed (57 %), modification of treatment duration (21 %), antibiotic spectrum narrowing (8 %), dose adjustment (7 %), and route modification (3 %).

The pandemic further bolstered preserving previously established stewardship achievements. Several AMS programmes implemented prior to COVID-19, including the Antibiotic Review Kit (ARK) and the Treat Antibiotics Responsibly, Guidance, Education, Tools (TARGET) toolkit, proved safe and effective in discontinuing antibiotics during acute hospital care (28). These findings highlight the need for sustained stewardship and its integration into future pandemic planning.

AMS implementation was strengthened through multidisciplinary collaboration involving pharmacists, infectious disease specialists, and microbiologists. Clinical pharmacists emerged as particularly important members of AMS teams during COVID-19. They ensured continuity of pharmaceutical supply, participated in the development and revision of COVID-19-specific treatment guidelines, and supported pandemic response systems by adapting existing stewardship programmes (19). Pharmacists also contributed through prospective audit and feedback activities, antimicrobial review, dose optimisation, de-escalation support, therapeutic drug monitoring and education of healthcare professionals regarding rational antibiotic use. Their unique expertise enabled tailored recommendations for appropriate antimicrobial prescribing and contributed significantly to minimising AMR development.

### Antibiotic prescribing patterns after COVID-19 vaccination

The introduction of large-scale COVID-19 vaccination programmes lowered disease severity, hospitalisation rates, and intensive care unit admissions, which likely contributed to reducing unnecessary antibiotic exposure. Jorgensen et al. (34) report a significant reduction in outpatient antibiotic prescribing, particularly of antibiotics commonly prescribed for respiratory tract infections, while no significant effect was observed for antibiotic treatment of urinary tract infections. One meta-analyses (35) reports 34 % lower odds of antibiotic use (OR: 0.662; 95 % CI: 0.540–0.811) in vaccinated COVID-19 patients.

The available evidence on the association between COVID-19 vaccination and antibiotic prescribing is summarised in Table 2.

**Table 2:** Impact of COVID-19 vaccination on antibiotic prescribing patterns

**Population**	**Main outcome**	**Effect of COVID-19 vaccination on antibiotic use**	**Ref.**
Older adults receiving COVID-19 vaccination	Antibiotic prescribing after vaccination	COVID-19 vaccination was associated with significantly reduced antibiotic prescribing, particularly for respiratory infections (OR 0.961; 95 % CI 0.953–0.968)	34
134,022 COVID-19 patients from 8 studies	Antibiotic use among COVID-19 patients	COVID-19 vaccination was associated with a 34 % reduction in the odds of antibiotic use (OR 0.662; 95 % CI 0.540–0.811)	35

### One Health implications of antibiotic overuse during the pandemic

Self-medication and uncontrolled access to antibiotics during the pandemic may have intensified global resistance pressure, particularly in low- and middle-income countries, increasing the risk of the rapid global spread of resistant microorganisms (27).

The implications of such antibiotic overuse are particularly evident in the disruption of the gut microbiome and increased incidence of CDI recorded among hospitalised elderly COVID-19 patients (12).

Furthermore, exposure to broad-spectrum antibiotics accelerated the emergence of multidrug-resistant organisms. One study (17) reported carbapenem resistance in hospital and intensive care settings in more than 69 % of Gram-negative isolates – *Acinetobacter baumannii*, *Klebsiella pneumoniae*, and *Pseudomonas aeruginosa* in particular. Widespread use of carbapenems, cephalosporins, and fluoroquinolones during the pandemic has raised serious concerns about long-term environmental effects and the amplification of AMR reservoirs (28).

Yet the consequences of excessive antibiotic use extend beyond antimicrobial resistance, as it has been associated with increased healthcare costs, adverse drug reactions, and prolonged hospitalisation (16). One study (21) reported an increase in healthcare costs from about $18,000 to $45,000, excluding staffing and other direct or indirect costs. Moreover, increased antimicrobial consumption must have strained global pharmaceutical supply chains and contributed to medication shortages worldwide (19).

One Health consequences, however, extend beyond patients and healthcare institutions, as antimicrobial resistance crosses human, animal, and environmental compartments (36). Antibiotics and their active metabolites are frequently excreted in urine and faeces and subsequently enter municipal wastewater and hospital effluents, whose residues cannot be completely eliminated by wastewater treatment and therefore persist in aquatic environments, facilitating interactions between human and environmental microbial communities (37, 38). Although direct evidence linking COVID-19-associated antibiotic consumption to subsequent environmental AMR remains limited, these established mechanisms call for the integration of wastewater surveillance, pharmaceutical waste management, and environmental AMR monitoring into broader One Health stewardship strategies (36,37,38).

### Diagnostic stewardship and rapid molecular diagnostics

Rapid molecular diagnostics support antimicrobial stewardship by facilitating earlier differentiation between bacterial and viral pneumonia. One study in critically ill COVID-19 patients (39), reported an 89.3 % sensitivity and 99.1 % specificity of a polymerase chain reaction diagnostic test that prompted initiation or modification of antibiotic therapy in 15 % and discontinuation in 28 % of episodes.

### The effects of infection prevention and non-pharmaceutical interventions on antimicrobial resistance

The pandemic produced several epidemiological effects that temporarily reduced antimicrobial demand. Non-pharmaceutical interventions, including social distancing, mask use, and better hand hygiene were associated with marked drops in viral and invasive bacterial infections (40, 41). These measures may also have reduced transmission of some healthcare-associated pathogens and mitigated the expected consequences of increased antibiotic exposure in certain settings (12).

In contrast, the widespread use of disinfectants and biocidal agents may also have contributed to selective pressure and potential cross-resistance among some microorganisms (42). These opposite effects further demonstrate that AMR trends cannot be understood from antibiotic consumption data alone and call for maintaining IPC measures alongside AMS during future health emergencies.

### Implications for future research and pandemic preparedness

What we have learned from the COVID-19 pandemic is that future AMS strategies should focus not only on reducing antibiotic prescribing but also on improving its quality and timing. Priority should be given to rapid and accessible microbiological diagnostics, structured reassessment of empirical therapy, or early discontinuation when bacterial infection is not confirmed, optimisation of treatment duration, restriction of unnecessary broad-spectrum agents, and to the application of multidisciplinary AMS infrastructure during public health emergencies (17, 19, 25, 27, 29, 32).

Future research should evaluate which stewardship interventions remain most effective under crisis, how diagnostic tools can best distinguish viral disease from bacterial co-infection in severely ill patients, and whether reductions in antibiotic exposure translate into measurable long-term reductions in AMR. Additional prospective evidence regarding the effect of vaccination on antibiotic prescribing and its environmental consequences would help future pandemic responses to balance the immediate need for safe empirical treatment against the long-term need to preserve antimicrobial effectiveness.

### Study limitations

This narrative review is limited by an inherent selection bias. The included studies may reflect early clinical practices that have changed since, whereas their designs, patient populations, and definitions of bacterial co-infection vary to such an extent that makes direct comparisons and synthesis nearly impossible. These limitations highlight the need for more high-quality, systematic studies to better understand the relationship between COVID-19 and antimicrobial resistance.

## CONCLUSION

The pandemic has revealed a large mismatch between the relatively low prevalence of bacterial co-infection and the widespread use of antibiotics, broad-spectrum in particular, among patients with COVID-19. Even though it boosted antimicrobial selection pressure, the relationship between antibiotic use and changes in AMR is not straightforward and involves multiple factors, including infection prevention and control, healthcare system pressures, diagnostic capacity, and antimicrobial stewardship activities.

Our review, however, provides some guidance as to how to maintain and strengthen antimicrobial stewardship for future public health emergencies through rapid microbiological diagnostics, timely reassessment of empirical therapy, appropriate de-escalation, optimisation of treatment duration, and avoidance of unnecessary broad-spectrum antibiotics. Robust infection prevention and control measures and continuous surveillance of antimicrobial consumption and resistance are equally essential.

Future preparedness strategies should adopt a One Health perspective, integrating clinical antimicrobial stewardship with public health and environmental surveillance. The experience of COVID-19 demonstrates that preserving antimicrobial effectiveness during future health emergencies will require resilient stewardship programmes, reliable diagnostic capacity, multidisciplinary collaboration, and evidence-based antimicrobial prescribing, even under conditions of substantial healthcare system pressure.
